# Supplementary material for: The causal role of circulating inflammatory markers in osteoporosis: a bidirectional Mendelian randomized study
Source: Front Immunol. 2024 Jul 18;15:1412298. doi: 10.3389/fimmu.2024.1412298 (PMC11291241; doi:10.3389/fimmu.2024.1412298)
Supplement: Supplementary file 1 [file DataSheet_1.zip › supplementary material/OSTEOPOROSIS-CIM/GCST90274843.tsv.gz_forest.pdf]

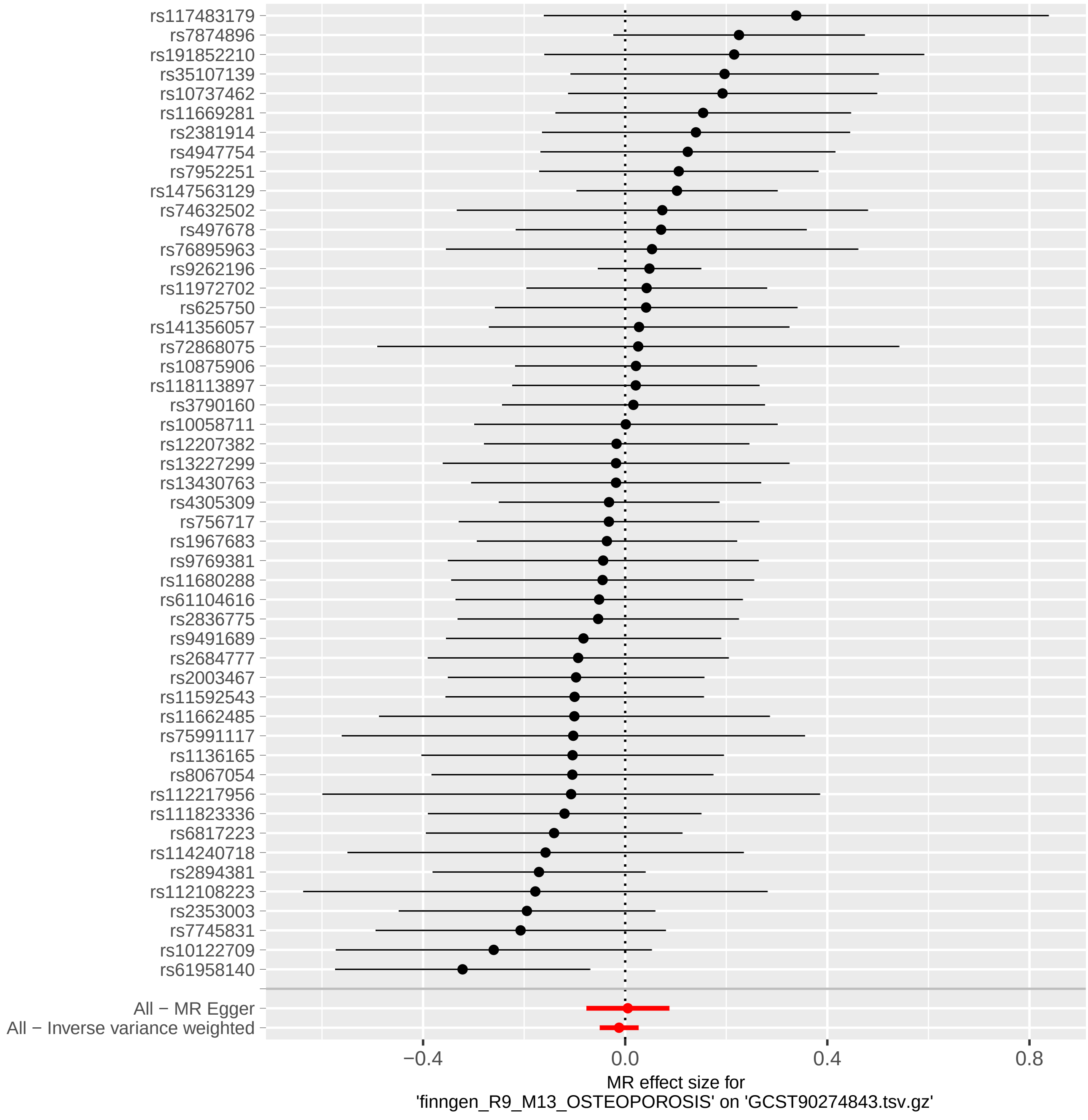
rs117483179
rs7874896
rs191852210
rs35107139
rs10737462
rs11669281
rs2381914
rs4947754
rs7952251
rs147563129
rs74632502
rs497678
rs76895963
rs9262196
rs11972702
rs625750
rs141356057
rs72868075
rs10875906
rs118113897
rs3790160
rs10058711
rs12207382
rs13227299
rs13430763
rs4305309
rs756717
rs1967683
rs9769381
rs11680288
rs61104616
rs2836775
rs9491689
rs2684777
rs2003467
rs11592543
rs11662485
rs75991117
rs1136165
rs8067054
rs112217956
rs111823336
rs6817223
rs114240718
rs2894381
rs112108223
rs2353003
rs7745831
rs10122709
rs61958140
All − MR Egger
All − Inverse variance weighted
−0.4
0.0
0.4
0.8
MR effect size for
'finngen_R9_M13_OSTEOPOROSIS' on 'GCST90274843.tsv.gz'
